# Efficient recombinant expression and secretion of a thermostable GH26 mannan endo-1,4-β-mannosidase from *Bacillus licheniformis *in *Escherichia coli*

**DOI:** 10.1186/1475-2859-9-20

**Published:** 2010-04-11

**Authors:** Chomphunuch Songsiriritthigul, Bancha Buranabanyat, Dietmar Haltrich, Montarop Yamabhai

**Affiliations:** 1 Synchrotron Light Research Institute (Public Organization), 111 University Avenue, Nakhon Ratchasima, Thailand; 2 School of Biotechnology, Institute of Agricultural Technology, Suranaree University of Technology, 111 University Avenue, Nakhon Ratchasima, Thailand; 3 BOKU, University of Natural Resources and Applied Life Sciences, Vienna, Austria

## Abstract

**Background:**

Mannans are one of the key polymers in hemicellulose, a major component of lignocellulose. The Mannan endo-1,4-β-mannosidase or 1,4-β-D-mannanase (EC 3.2.1.78), commonly named β-mannanase, is an enzyme that can catalyze random hydrolysis of β-1,4-mannosidic linkages in the main chain of mannans, glucomannans and galactomannans. The enzyme has found a number of applications in different industries, including food, feed, pharmaceutical, pulp/paper industries, as well as gas well stimulation and pretreatment of lignocellulosic biomass for the production of second generation biofuel. *Bacillus licheniformis *is a Gram-positive endospore-forming microorganism that is generally non-pathogenic and has been used extensively for large-scale industrial production of various enzymes; however, there has been no previous report on the cloning and expression of mannan endo-1,4-β-mannosidase gene (*manB*) from *B. licheniformis*.

**Results:**

The mannan endo-1,4-β-mannosidase gene (*manB*), commonly known as β-mannanase, from *Bacillus licheniformis *strain DSM13 was cloned and overexpressed in *Escherichia coli*. The enzyme can be harvested from the cell lysate, periplasmic extract, or culture supernatant when using the pFLAG expression system. A total activity of approximately 50,000 units could be obtained from 1-l shake flask cultures. The recombinant enzyme was 6 × His-tagged at its C-terminus, and could be purified by one-step immobilized metal affinity chromatography (IMAC) to apparent homogeneity. The specific activity of the purified enzyme when using locust bean gum as substrate was 1672 ± 96 units/mg. The optimal pH of the enzyme was between pH 6.0 - 7.0; whereas the optimal temperature was at 50 - 60°C. The recombinant β-mannanase was stable within pH 5 - 12 after incubation for 30 min at 50°C, and within pH 6 - 9 after incubation at 50°C for 24 h. The enzyme was stable at temperatures up to 50°C with a half-life time of activity (τ1/2) of approximately 80 h at 50°C and pH 6.0. Analysis of hydrolytic products by thin layer chromatography revealed that the main products from the bioconversion of locus bean gum and mannan were various manno-oligosaccharide products (M2 - M6) and mannose.

**Conclusion:**

Our study demonstrates an efficient expression and secretion system for the production of a relatively thermo- and alkali-stable recombinant β-mannanase from *B. licheniformis *strain DSM13, suitable for various biotechnological applications.

## Background

The Mannan endo-1,4-β-mannosidase or 1,4-β-D-mannanase (EC 3.2.1.78), commonly named β-mannanase, is an enzyme that can catalyze random hydrolysis of β-1,4-mannosidic linkages in the main chain of β-1,4-mannans, glucomannans and galactomannans; thus it transforms the abundant heteromannans to manno-oligosaccharides [1,2] and a small amount of mannose, glucose and galactose [3]. Mannan endo-1,4-β-mannosidases are produced by a number of plants, bacteria, fungi, and by various invertebrates. The enzyme has found a number of applications in different sectors [4], including food, feed, pharmaceutical, and pulp/paper industries, gas well stimulation [1], as well as pre-treatment of lignocellulosic biomass for the production of second generation biofuel [2]. The application of mannan endo-1,4-β-mannosidase for the production of prebiotic manno-oligosaccharides from cheap agricultural by-products such as copra has recently gained significant interests [5-8].

*Bacillus licheniformis *is a Gram-positive endospore-forming microorganism that belongs to the *B. subtilis *group of the genus *Bacillus*. It is generally non-pathogenic and has been used extensively for large-scale industrial production of exoenzymes such as subtilisins or amylase, and the antibiotic bacitracin [9]. Recently, the genome of *B. licheniformis *strain DSM13 has been reported and it was revealed that it contains many new genes of potential interest for biotechnological applications [10]. So far, there has been no previous report on the cloning and expression of mannan endo-1,4-β-mannosidase gene (*manB*) from *B. licheniformis*; however, there were some preliminary reports on the property of native enzymes [11,12]. In this work *manB *from *B. licheniformis *strain DSM13, which has been used extensively in industry, was cloned and overexpressed using an *Escherichia coli *expression system [13]. The recombinant enzyme was highly expressed and efficiently secreted into the periplasmic space and subsequently into the culture medium. Amino acid sequence analysis revealed that the enzyme belongs to the glycosyl hydrolase family GH26 [14]. The properties of the recombinant enzyme are also reported, and our results demonstrate that recombinant mannan endo-1,4-β-mannosidase from *B. licheniformis *is thermo- and alkali-stable, and thus suitable for various industrial applications.

## Results

### Cloning and expression of mannan endo-1,4-β-mannosidase from *Bacillus licheniformis*

**Figure 1:**
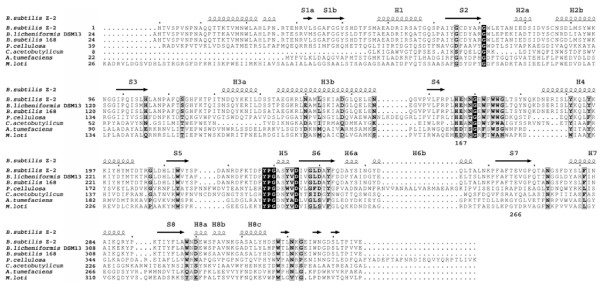
**Amino acid sequence alignment of ManB from *B. licheniformis *and other mannan endo-1,4-β-mannosidases belonging to glycosyl hydrolase family 26 (GH26)**. α-Helices are displayed as squiggles; β-strands are rendered as arrows. The eight β-strands forming the core of the TIM-barrel are referred as S1 to S8, whereas the eight α-helices connecting these β-strands are referred to as H1 to H8. A white character in a box indicates strict identity, while a black character in a frame indicates similarity across a group. The catalytic glutamate residues are located at position 167 and 266 of β-mannanase from *B. subtilis *Z-2 [16]. Multiple sequence alignment was done by CLUSTAL W [36] followed by ESPript [37] to display the secondary structure of the β-mannanase from *B. subtilis *Z-2, 2QHA. The similarity of different enzymes is shown as % identity, based on the sequence of *B. licheniformis *DSM13 (100%); B. *subtilis Z-2 *(81.90%); *B. subtilis *168 (81.90%); *P. cellulosa *(19.90%); *C. acetobutylicum *(15.27%); *A. tumefaciens *(13.82%) and *M. loti *(11.08%). Key: *B. subtilis *Z-2 (Bacillus subtilis Z-2, PDB code; 2QHA); *B. licheniformis *DSM 13 (*Bacillus licheniformis *DSM 13, NCBI accession number NC006322); *B. subtilis *168 (*Bacillus subtilis *subsp. subtilis str. 168, NCBI accession number NC000964); *P. cellulosa *(*Pseudomonas cellulose*, NC010995); *C. acetobutylicum *(*Clostridium acetobutylicum *str. ATCC 824, NC003030); *A. tumefaciens *(*Agrobacterium tumefaciens *str. C58, NCBI accession number NC003063) and *M. loti *(*Mesorhizobium loti *MAFF303099, NCBI accession number, NC002678).

The oligonucleotide primers for cloning of the *manB *gene encoding mannan endo-1,4-β-mannosidase from *B. licheniformis *DSM13 were designed from its complete genomic database, according to the DNA sequence of gene *ydhT *(NCBI accession number NC006322), encoding a hypothetical protein similar to mannan endo-1,4-beta-mannosidase. The gene was cloned into the pFLAG expression vector such that the hypothetical native signal peptide was replaced with the *E. coli *OmpA signal peptide included in this vector. This allows the secretion of the recombinant enzyme into the periplasmic space and subsequently into culture broth. In addition, the DNA sequence encoding a hexahistidine together with a stop codon was incorporated into the reverse primers to create a His-tagged fusion enzyme to facilitate further purification. The *manB *gene was under control of the *tac *promoter and could be induced for high expression using Isopropyl β-D-1-thiogalactopyranoside (IPTG). Amino acid sequence analysis revealed that the mannan endo-1,4-β-mannosidase from *B. licheniformis *has a theoretical molecular mass of 41 kDa, and belongs to glycosyl hydrolase family GH26, according to the CAZy (CArbohydrate-Active EnZymes) databank [15]. This family is a member of clan Glyco hydro tim or TIM barrel glycosyl hydrolase (GH) superfamily, which comprises 26 members, including α-amylase and cellulase. The deduced amino acid sequence alignment of *B. licheniformis *mannan endo-1,4-β-mannosidase with other bacterial β-mannanases from family 26 is shown in Fig. 1. The enzyme shows the classical TIM (β/α)8-barrel architecture. The catalytic domains of GH26 members are located at the C-terminus, and conserved amino acid residues of this glycosyl hydrolase family are also shown in Fig. 1. The mannan endo-1,4-β-mannosidase from *B. licheniformis *is highly similar to ManB from *B. subtilis *Z-2 [16] and *B. subtilis *strain 168 [17] with 82% identity, whereas its similarity to GH26 β-mannanases from other bacterial species is significantly less (11-20% identity).

### Expression and secretion of mannan endo-1,4-β-mannosidase

**Figure 2:**
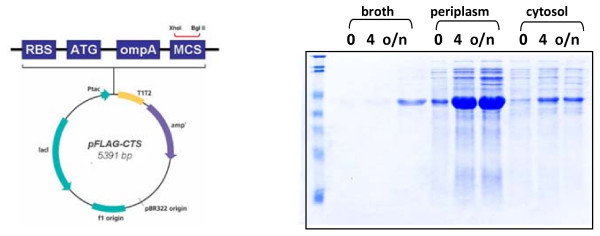
**Expression and secretion of recombinant mannan endo-1,4-β-mannosidase (ManB) from *B. licheniformis***. A) The pFLAG-CTS system (Sigma) was used for the expression of recombinant β-mannanase. Enzyme expression was under the control of *tac *promoter, which can be induced for overexpression by IPTG. The vector contains the ampicillin resistant gene, and a hexahistidine tag was incorporated C-terminally. The mature enzyme was fused to the *E. coli *OmpA signal peptide (shown in a box) for secretion into the periplasmic space. B) Cells were grown until OD_600 _reach ~1.0 before IPTG was added to a final concentration of 1 mM, and incubation continued at 28°C. Samples were taken at 0, 4 h, and overnight (20 h, o/n) after induction with IPTG. Culture supernantant, periplasmic and cytosolic fractions were prepared as described in Material and Method. Approximately equal amounts of total protein in the periplasmic and cytosolic fraction were loaded onto each lane.

The recombinant mannan endo-1,4-β-mannosidase produced in this study was fused with the *E. coli *OmpA signal peptide and thereby could be efficiently secreted into the periplasmic space and culture medium as has been previously reported for this expression system and various secreted *Bacillus*-derived enzymes [13]. Both the efficient expression and extracellular location after induction with IPTG are evident from Fig. 2. At 4 h after induction, a large fraction of the recombinant enzyme was accumulated in the periplasmic space, and after inducing overnight, more enzymes could be found in the culture medium than in the periplasmic space. Comparison of the yield of recombinant *B. licheniformis *mannan endo-1,4-β-mannosidase in different compartments at various induction conditions in shake flask is given in Table 1. The highest specific activity could be obtained from perisplasmic extract after induction with 0.5 mM IPTG overnight, whereas the largest total activity could be obtained from the three fractions after induction with 1 mM IPTG for 4 h. Nevertheless, other conditions yield only slightly different results. Routinely, we obtained 45 - 50,000 U of total mannan endo-1,4-β-mannosidase activity from a 1-l shake flask culture. To prepare the enzyme for purification and analysis in the next step, we preferred to use the cytoplasmic and especially the periplasmic extract, as the enzyme was highly concentrated, facilitating the subsequent affinity purification step.

**Table 1:** Yield of recombinant *B. licheniformis *mannan endo-1,4-β-mannosidase in different cell compartments of *E. coli*

Induction	Culture supernatant		Periplasmic Space		Cytoplasm		Total	
	
	4 hr	Ovn	4 hr	Ovn	4 hr	Ovn	4 hr	ovn
***0.1 mM IPTG***								
Total Activity (U/L)	8,659	15,076	13,509	9,983	15,189	24,502	**37,357**	**49,561**
Total Protein (mg/L)	147	170	44.5	17.8	186	163	**378**	**351**
Sp. Activity (U/L)	58.8	89.0	304	560	81.7	150	**99.0**	**141**

***0.5 mM IPTG***								
Total Activity (U/L)	8,437	14,171	19,649	13,488	10,589	24,480	**38,675**	**52,139**
Total Protein (mg/L)	137	156	45.0	20.5	137	199	**319**	**376**
Sp. Activity (U/L)	61.7	91.1	437	660	77.3	123	**121**	**139**

***1.0 mM IPTG***								
Total Activity (U/L)	13,561	14,402	17,647	13,232	24,502	26,632	**55,710**	**54,266**
Total Protein (mg/L)	153	175	39.5	20.2	163	168	**356**	**363**
Sp. Activity (U/L)	88.9	82.4	447	656	150	159	**157**	**149**

The typical yield of the overexpression of recombinant mannan endo-1,4-β-mannosidase from *B. licheniformis *in *E. coli *grown in 1-liter shaken flask cultures is reported. Cells were cultivated and enzymes from various compartments were harvested after induction for 4 and 20 h as described in Material and Method.

### Enzyme purification and assay

**Figure 3:**
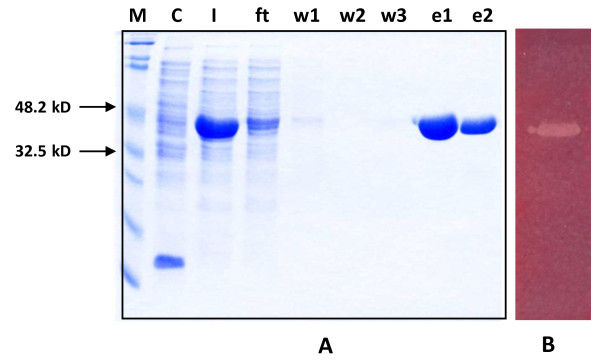
**Coomassie staining and zymogram analysis of purified recombinant mannan endo-1,4-β-mannosidases from *B. licheniformis***. SDS-PAGE analysis of purified recombinant β-mannanase is shown in panel A. M, marker; C, control *E. coli *lysate; I, input (crude extract); Ft, flow through; w1-3, wash 1-3; e1-2, enzyme from 1st and 2nd elution. Panel B illustrates zymogram analysis of the purified enzyme. Only 1/1000 of the amount used in the left panel was loaded onto the gel. White bands indicate mannan endo-1,4-β-mannosidase activity.

The crude enzyme from the periplasmic extract was used for affinity purification on Ni-NTA agarose. The enzyme could be purified to apparent homogeneity using this one-step purification protocol as shown by SDS-PAGE analysis (Fig. 3, panel A). Mannan endo-1,4-β-mannosidase activity of the purified enzyme was shown by in-gel activity staining (Fig. 3, panel B) as well as by the standard β-mannanase assay. Recombinant ManB showed a molecular mass of approximately 45 kDa on SDS-PAGE, confirming the theoretical mass of 41 kDa. The specific activity of the homogenous enzyme was 1672 ± 96 U/mg under the standard assay conditions. We routinely obtained a total of approximately 40,000 U of purified enzyme (equivalent to < 25 mg) from 1-l cultures.

### Effect of pH and temperature

**Figure 4:**
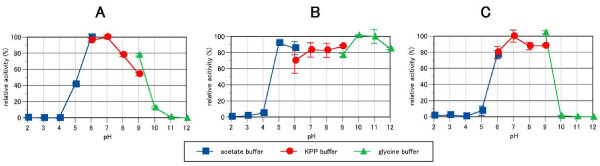
**Effect of pH on the activity (A) and stability (B, C) of *B. licheniformis *mannan endo-1,4-β-mannosidase**. The optimal pH was determined at 50°C using 0.5% LBG in 50 mM of different buffers (A). The pH stability was determined by measuring the remaining activity after incubation at various pH values at 50°C for 30 min (B) and 24 h (C). The buffers used were acetate buffer (black square) from pH 2 - 6; potassium phosphate buffer; KPP (black circle) from pH 6 - 9; and glycine buffer (black triangle) from pH 9 - 12.

**Figure 5:**
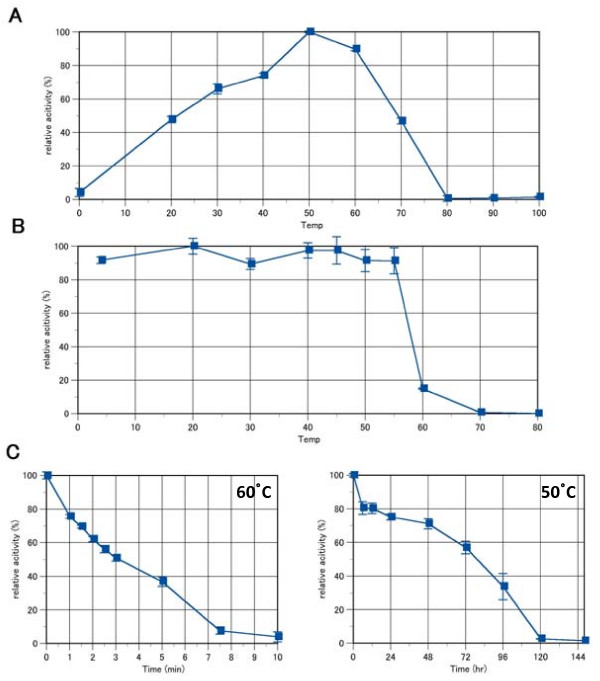
**Effect of temperature on activity (A) and stability (B, C) of *B. licheniformis *mannan endo-1,4-β-mannosidase**. The optimal temperature was determined using 0.5% LBG in 50 mM citrate buffer, pH 6.0 (A). The temperature stability was determined by measuring the remaining activity after incubation without substrate at various temperatures at pH 6.0 for 30 min, and measuring the residual activity using the standard assay (B). Panel C illustrates the remaining enzyme activity after incubation at 60°C (left) and 50°C (right) at various time points.

The optimal pH of mannan endo-1,4-β-mannosidase activity from *B. licheniformis *was at pH 6.0 - 7.0 (Fig. 4, panel A). Notably, the enzyme shows a significant activity up to pH 9.0, and is more active at this pH when using glycine buffer than potassium phosphate buffer. The enzyme was stable within pH 5 - 12 after incubation for 30 min at 50°C (Fig. 4, panel B), and within pH 6 - 9 after incubation at 50°C for 24 h (Fig. 4, panel C). The optimal temperature for ManB activity was 50 - 60°C for the 5-min assay (Fig. 4, panel A). The enzyme was stable up to 55°C after incubation for 30 min at pH 6.0 (Fig. 5, panel B). In addition, it showed a half-life time of activity, τ1/2 of approximately 80 h at 50°C and pH 6.0, while τ1/2 decreased considerably to only 3 min at 60°C (Fig. 5C).

### Substrate specificity and kinetic parameters

The relative activity of ManB from *B. licheniformis *for various substrates was determined as shown in Table 2. The enzyme exhibited highest activity on glucomannan prepared from konjac followed by pure 1,4-β-D-mannan and the galactomannan locust bean gum (LBG). The activity of the enzyme with highly substituted galactomannan from guar gum and copra meal was negligible when using the standard assay. However, we found that partial hydrolysis of copra meal after incubation occurred after incubation of this substrate with the enzyme for 2 to 3 days (data not shown).

**Table 2:** Substrate specificity of *B. licheniformis *mannan endo-1,4-β-mannosidase

Substrate	Relative activity (%)
Glucomannan (konjac)	219

1,4-β-D-Mannan	166

Locust bean gum	100

Guar gum	nd

Copra meal	nd

The activity of mannan endo-1,4-β-mannosidase from *B. licheniformis *was determined under standard assay conditions using each substrate at a concentration of 5 g/l. The relative activity with the standard substrate locust bean gum (high viscosity) was defined as 100%.

nd: no apparent activity at standard assay condition was detected.

Furthermore, the kinetic constants for the hydrolysis of selected substrates were determined. Because of the extremely high viscosity of LBG solutions, especially at higher concentrations necessary for the determination of the kinetic constants, low-viscosity LBG was prepared by partial hydrolysis [18] and used as a substrate in addition to glucomannan from konjac and pure 1,4-β-D-mannan. When present in saturating concentrations, low-viscosity LBG was the preferred substrate as judged both from the highest turnover number kcat and specificity constant kcat/Km (Table 3).

**Table 3:** Kinetic parameters of the purified mannan endo-1,4-β-mannosidase

Substrate	V_max_ (μmol min^-1^mg^-1^)	K_m_ (mg ml^-1^)	*k*_*cat*_ (s^-1^)	*k*_*cat*_/K_m_ (mg^-1^s^-1^ml)
Glucomannan	30,400	14.9	21,000	1,410

LBG(low viscosity)	45,300	17.5	31,200	1,790

β-D-Mannan	26,400	15.2	18,200	1,200

### Product analysis by thin-layer chromatography

**Figure 6:**
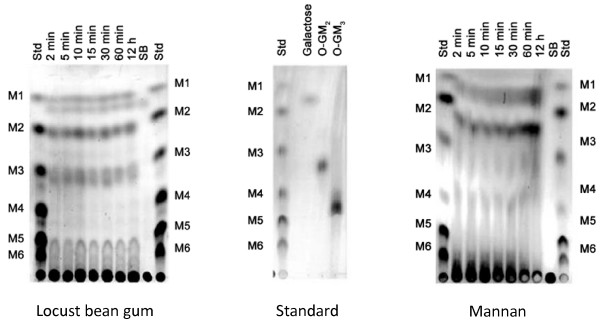
**Thin layer chromatography analysis of hydrolysis products using LBG and Mannan as substrates**. Products from LBG and mannan hydrolysis at various time points are illustrated. Std: a standard mixture of M1 - M6; 2 min, 5 min, 10 min, 15 min, 30 min, 60 min, 12 h are the reaction products after incubation at 2, 5, 10, 15, 30 and 60 min, and 12 h respectively; SB: substrate blank.

**Figure 7:**
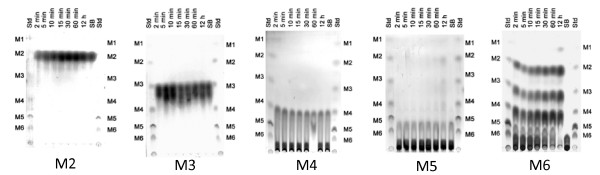
**Thin layer chromatography analysis of hydrolysis products using various manno-oligosaccharides as substrates**. Hydrolysis products when using mannobiose (M2), mannotriose (M3), mannotetraose (M4), mannopentaose (M5) and mannohexaose (M6) as substrates are shown. Std: a standard mixture of M1 - M6; 2 min, 5 min, 10 min, 15 min, 30 min, 60 min, 12 h are the reaction products after incubation at 2, 5, 10, 15, 30 and 60 min, and 12 h respectively; SB: substrate blank.

Product analysis by TLC after hydrolysis of various substrates confirmed that the recombinant enzyme is indeed an endo-β-mannanase. Various manno-oligosaccharide products (M2 - M6) as well as mannose were found after enzymatic hydrolysis of locust bean gum and mannan (Fig. 6). When mannohexaose (M6) was used as a substrate (Fig. 7), the main products were M2, M3 and M4, suggesting random hydrolysis of this oligosaccharide. After extensive overnight digestion, mannose (M1) could be observed as well. Analysis of hydrolysis products when using different manno-oligosaccharides (M2 - M5) as substrates revealed that ManB from *B. licheniformis *cannot cleave mannobiose, mannotriose or mannotetraose, whereas mannopentaose was hydrolysed only after extensive incubation overnight, generating M2 and M3 as products (Fig. 7).

## Discussion

*B. licheniformis *strain DSM13 was used as the source for the isolation of the mannan endo-1,4-β-mannosidase gene, *manB *since this strain has been used extensively for large-scale production of various industrial enzymes including serine protease (subtilisin) or α-amylase [9]. The genome of strain DSM13 has recently been sequenced, and a number of new genes of potential biotechnological applications have been identified [10]. The mannan endo-1,4-β-mannosidase gene was cloned by PCR cloning, using primers designed from the published genome database. This is the first report on the cloning, expression, and characterization of recombinant mannan endo-1,4-β-mannosidase from *B. licheniformis*. Other reports on recombinant *Bacillus *mannan endo-1,4-β-mannosidases were dealing with enzymes from *B. subtilis *[16,19-23]. *B. stearothermophilus *[24], and *B. circulans *[25].

Mannan endo-1,4-β-mannosidases can be classified into two distinct families, glycosyl hydrolase (GH) family 5 and 26, based on amino acid sequence similarities and hydrophobic cluster analysis [14]. Family GH5 was formerly known as cellulase family A and encompasses diverse enzymes [26], whereas glycosyl hydrolase family 26 comprises only members with mannan endo-1,4-β-mannosidase (EC 3.2.1.78) and β-1,3-xylanase (EC 3.2.1.32) activities [14]. Amino acid sequence analysis of mannan endo-1,4-β-mannosidase from *B. licheniformis *revealed that the enzyme belongs to family GH26. In addition, we also cloned and expressed the mannan endo-1,4-β-mannosidase gene (*manB*) from *B. licheniformis *strain DSM 8785. The two enzymes have only one amino acid different, and the properties of these two heterologously expressed recombinant enzymes are identical (data not shown).

The expression and production of the recombinant mannan endo-1,4-β-mannosidase reported here is based on a previously published *E. coli *expression system [13]. The mature mannan endo-1,4-β-mannosidase gene was fused to the *E. coli *ompA signal sequence and is under the control of *tac *promoter. Thus, the enzyme could be efficiently secreted, and harvested from the culture medium, periplasm, or cell lysate fraction, depending on the culture condition. When the gene was induced for over-expression by 1 mM IPTG for 3 - 4 h, we routinely obtained about 25 mg of recombinant enzyme from the cytoplasmic and periplasmic extracts of 1-liter cultures, which contain more than 40,000 units of purified enzyme. Under the induction with IPTG, a significant fraction of the enzyme was still found in the cytosol. This could indicate that the over-expressed enzyme possibly saturates the bacterial secretion system [27]. It should be mentioned that no optimization aiming at increased enzyme yields was performed. Thus, by applying optimized culture and induction conditions together with a suitable fermentation strategy, considerably higher recombinant protein yields can be expected. Thus, our expression system is highly efficient for expression of bacterial β-mannanses and should be applicable for other enzymes as well. More importantly, the extracellular location of the enzyme might be of interest for large-scale cultivations as it circumvents the necessity of cell disruption.

Mannan endo-1,4-β-mannosidases are active on various mannans and substituted mannans, but display negligible to low activity towards other plant cell wall polysaccharides [3,28]. The enzymes randomly hydrolyse β-1,4-linkages in diverse substrates such as pure mannans, galactomannans, glucomannans and galactoglucomannans [4]. In this study, we found that *B. licheniformis *ManB shows the highest relative activity for glucomannan prepared from konjac followed by pure low-molecular mass 1,4-β-D-mannan of DP (degree of polymerization) < 15 and high-viscosity (high molecular mass) locust bean gum. However, we were not able to detect notable activity for guar gum and copra meal using the standard assay of 5-min incubation. Based on the kinetic characterization and judged from the specificity constant kcat/Km, the galactomannan locust bean gum (low viscosity) is the preferred substrate, however the differences in the specificity constant are not very pronounced when compared to konjac glucomannan and pure mannan. Apparently, *B. licheniformis *ManB prefers soluble and low-substituted mannan substrates. This is evident from a comparison of the relative activity on soluble LBG, a galactomannan from *Ceratonia siliqua *with a mannose-to-galactose ratio of 4:1, and soluble guar gum, a galactomannan from *Cymopsis tetragonoloba *with a mannose-to-galactose ratio of 2:1 [1]. While the former is a good substrate, the activity on the latter is negligible during the 5-min standard assay. Similarly, activity on copra mannan, an insoluble galactomannan with a very low degree of galactosyl substitution, is very low [29].

There have been a number of reports on the characterization of mannan endo-1,4-β-mannosidases, both native and recombinant, from various organisms as summarized in Additional file 1. The pH and temperature optima as well as the stability of the enzymes are clearly varying, depending on the sources of the enzymes. Typically, the enzymes from non-bacterial sources show lower pH and temperature optima as well as lesser stability (See Additional file 1). The specific activity (from 3.8-8300 U/mg) and kinetic parameters (Km ranging from 0.3-10.2, Vmax from 3.8-2000) of the mannan endo-1,4-β-mannosidases from various sources, when using LBG as a substrate, vary greatly as shown in Additional file 1. This obviously reflects differences in the structure of the enzymes, for example highly thermostable mannan endo-1,4-β-mannosidase tend to have lower specific activity compared to their mesophilic counterparts [24,30,31]. In this respect, the *B. licheniformis *ManB described in our report is characterized by a very high specific activity of 1672 U/mg as well as by a relatively high stability. However, when comparing different mannan endo-1,4-β-mannosidases it is important to note that locust bean gum, which is a standard substrate for measuring mannan endo-1,4-β-mannosidase activity, is highly viscous and difficult to prepare. It can be assumed that the large discrepancy of enzyme activity in some of the reports can in part result from various techniques used in substrate preparation. For example we were not able to estimate with confidence the kinetic parameters when using high-viscosity, commercial LBG as a substrate. Thus, only the kinetic parameters when using low-viscosity LBG, low-viscosity glucomannan from konjac, and β-mannan are reported here.

TLC analysis of hydrolysis products confirmed that recombinant *B. licheniformis *mannanse is an endo-mannanase, which can efficiently and randomly cleave higher molecular weight mannans containing more than six mannose monomers. The enzyme could only cleaved mannopentaose after an extended incubation for 12 h and had no detectable activity against mannobiose, -triose or -tetraose. This property suggests that this enzyme could be applicable for the generation of prebiotic manno-oligosaccharides (MOS), as higher oligosaccharides formed will not be hydrolyzed further. Extensive hydrolysis of cheap and commercial available locust bean gum can therefore result in a mixture of MOS containing various oligosaccharides that may have a diverse prebiotic and anti-obesity [8] effects in different regions of the gut. Higher oligosaccharides are currently discussed as prebiotics with enhanced persistence that can reach more distal regions of the gut, and thus show their positive effect also in that region [32].

## Conclusion

Our results demonstrate an efficient system for expression and secretion of a relatively thermo- and alkali-stable β-mannanase from *B. licheniformis*, which is suitable for industrial applications. In addition, the expression and secretion system that is used in this study could be adopted for production of other enzymes as well.

## Methods

### Bacterial strains and culture conditions

*Bacillus licheniformis *DSM13 (ATCC 14580) was obtained from DSMZ; German Culture Collection of Microorganisms and Cell Cultures (Braunschweig, Germany). Cells were grown at 37°C and kept in M1 medium. *Escherichia coli *DH5α (Life Technologies) was used in the molecular cloning experiments, whereas *E. coli *Top10 (Invitrogen) was used for expression of the recombinant enzyme. The *E. coli *strains were grown in Luria broth (LB) containing 100 μg/ml of ampicillin for maintaining the plasmid.

### Molecular cloning of mannan endo-1,4-β-mannosidase from *Bacillus licheniformis*

The gene of the mature mannan endo-1,4-β-mannosidase was cloned by a PCR-based method. The primers B.liManfwXhoI: CTG TGC CTC GAG CAC ACA CCG TTT CTC CGG TG, and B.liManrv6HiBgl2: CTG TGC AGA TCT TCA ATG GTG ATG GTG ATG GTG TTC CAC GAC AGG CGT CAA AGA ATC GCC were used for PCR amplification of *manB*. These primers were designed using the published sequence from the genomic database of *B. licheniformis *DSM13 (NCBI accession number. NC006322, REGION: 739316740398), and were compatible with the *Xho*I and *Bgl*II cloning sites of pFLAG-CTS expression vectors (Sigma). The DNA encoding native signal peptides were omitted, and the gene of the mature enzyme was fused with the *E. coli *OmpA signal peptide instead in order to enable efficient secretion into the periplasm and culture media. In addition, DNA encoding a hexahistidine tag was incorporated into the reverse primers to generate 6 × His tagged recombinant enzymes for further purification. PCR reactions were performed according to the recommendations from the manufacture in a thermal cycler from MJ Research. Templates were prepared by boiling a single colony of *B. licheniformis *in 100 μl of water for 5 min, and 50 μl of that solution were directly used in the PCR reaction. The PCR reaction (total volume of 100 μl) consisted of 0.5 μM of primers, 0.2 mM dNTP, 3 units of *Pfu *DNA polymerase (Promega), and 10 × reaction buffer, provided by the manufacturer. The amplifications were done as follows: initial DNA denaturation at 95°C for 2 min; 30 cycles of denaturation at 95°C for 45 sec, annealing at 58°C for 1 min, extension at 72°C for 2.5 min, and a final extension at 72°C for 10 min. The PCR products were separated on 1% agarose gels containing ethidium bromide and visualized under a UV transilluminator. PCR products were purified using PCR purification kits (Qiagen, Germany). The PCR products were then cut with appropriate restriction enzymes (*Xho*I and *Bgl*II) and ligated into the pFLAG-CTS expression vector that has been cut with corresponding enzymes. The ligation reactions were transformed into *E. coli *DH5α. The DNA sequence and the integrity of the constructs were determined by automated DNA sequencing (Macrogen, Korea).

### Expression of recombinant enzymes

Freshly transformed *E. coli *Top 10 harbouring the recombinant *manB *gene was inoculated into 5 ml of LB broth containing 100 μg/ml of ampicillin at 37°C for 16 h. After that, 1 ml of overnight culture was inoculated into 250-1000 ml of LB broth containing 100 μg/ml ampicillin and grown at 37°C until the optical density at 600 nm reached ~1.0 - 1.5. Then, IPTG was added into the culture broth to a final concentration of 0.1 - 1.0 mM. The culture was subsequently incubated with vigorous shaking (250 rpm) at 26-28°C (room temperature) for 3 - 4 h. The culture was collected and chilled in an icebox for 5 min and centrifuged at 2,000 × g for 10 min at 4°C to separate cells and supernatant. To extract the periplasmic content, the cells were resuspended in 2.5 ml of cold (4°C) spheroplast buffer [100 mM Tris-HCl, pH 8.0, 0.5 mM EDTA, 0.58 M sucrose, and 20 μg/ml phenylmethylsulfonyl fluoride (PMSF)]. After incubation for 5 min on ice, bacterial cells were collected by centrifugation at 8,000 × g at 4°C for 10 min and re-suspended in 1-2 ml of ice-cold sterile water supplemented with 1 mM MgCl_2 _and incubated on ice for 5 minutes with frequent shaking. The supernatant of approximately 1 - 2 ml was then collected by centrifugation at 8,000 × g at 4°C for 15 min as the periplasmic fraction. To extract the cell lysate, the precipitated cells from the previous step were washed once with lysis buffer (50 mM Tris-HCl + 0.5 mM EDTA), resuspended in 1 - 2 ml of lysis buffer, and sonicated (Ultrasonic Processor; 60 amplitude, pulser 6 sec, for 2 min) on ice. The cell debris was then spun down at 8,000 × g and the supernatant was collected as the cell lysate.

### Purification of recombinant mannan endo-1,4-β-mannosidase

Immobilized metal affinity chromatography (IMAC) was used for purification of 6 × His-tagged recombinant β-mannanase by gravity-flow chromatography, using Ni-NTA Agarose according to the manufacturer protocol (Qiagen). The periplasmic extract was loaded onto a column and washed three times with increasing concentrations of imidazole of 5, 10 and 20 mM. The enzyme was then eluted by elution buffer containing 250 mM imidazole, and dialyzed using a dialysis membrane (Pierce Biotechnology, 10-kDa molecular-weight cutoff) to remove imidazole.

### Gel electrophoresis and zymogram analysis

Denaturing sodium dodecyl sulfate-polyacrylamide gel electrophoresis (SDS-PAGE) was performed according to the method of Laemmli [33], in a 12% (w/v) polyacrylamide gel. The protein samples were briefly heated (3 min) in the loading buffer at 100°C using a heat block (Eppendorf). Protein bands were visualized by staining with Coomassie brilliant blue R-250. The molecular weight markers were from Biorad.

A zymogram of mannan endo-1,4-β-mannosidase activities was generated by an in-gel activity assay using 0.25% locust bean gum as substrate, copolymerized with 10% (w/v) polyacrylamide. The enzyme samples were mixed with the loading buffer in the absence of reducing agent, and then applied onto a polyacrylamide gel. After electrophoresis, the gel was soaked in 2.5% Triton X-100 for 30 min at 4°C, and incubated in sodium phosphate buffer pH 7.0 at 50°C for 1 h. The gel was then rinsed with de-mineralized water, stained with 0.1% Congo red solution and gentle shaking for 20 min prior to destaining with 1 M NaCl for 20-30 min, and thereafter was placed in 5% acetic acid for 3 min (optional). Mannan endo-1,4-β-mannosidase activity was detected as clear zones against red (after staining with Congo red) or blue background (after soaking in 5% acetic acid).

### Protein determination

Protein concentration was determined by the method of Bradford [34] using bovine serum albumin as standard.

### Enzyme assays

Standard mannan endo-1,4-β-mannosidase activity was assayed using the dinitrosalicylic acid (DNS) method [35]. The substrate, 0.5% locust bean gum (Sigma), was dissolved in 50 mM sodium citrate buffer, pH 6.0 by homogenizing at 80°C, heated to the boiling point, cooled and stored overnight with continuous stirring. After that insoluble was removed by centrifugation. An appropriately diluted enzyme solution (0.1 ml) was incubated with 0.9 ml of the substrate solution at 50°C for exactly 5 min. The amount of reducing sugars liberated in the enzyme reaction was assayed by mixing 100 μl of the enzyme reaction with 100 μl DNS solution, heating at 100°C for 20 min, cooling on ice, and diluting with 300 μl of de-ionized water before measuring the absorbance at 540 nm. One unit of mannan endo-1,4-β-mannosidase activity is defined as the amount of enzyme that liberates 1 μmol of reducing sugar (using D-mannose as a standard) per minute under the experimental conditions given.

### Effect of pH and temperature on enzyme activity

The optimal pH of mannan endo-1,4-β-mannosidase activity was measured between pH 2.0 - 12.0 under standard assay condition, using three buffer systems (each 50 mM): sodium acetate (pH 2.0 - 6.0), potassium phosphate (pH 6.0 - 9.0), and glycine (pH 9.0 - 12.0). To determine the pH stability of mannan endo-1,4-β-mannosidase, enzyme samples were incubated at various pH values using the same buffer systems as above at 50°C for 30 min or 24 h, and then the remaining enzyme activity was measured under standard assay condition.

The temperature dependence of mannan endo-1,4-β-mannosidase activity was measured by incubating the enzyme samples with the substrate at temperatures ranging from 4 - 100°C in 50 mM citrate buffer pH 6.0. Thermal stability of the enzyme was determined by incubating enzyme samples in 50 mM citrate buffer, pH 6.0, at various temperatures ranging from 4 - 80°C for 30 min, then the remaining enzyme activity was measured under standard assay condition. In addition, the thermal inactivation kinetics at 50 and 60°C were determined in 50 mM citrate buffer, pH 6.0, by measuring the residual enzyme activity at certain time points assayed under standard condition.

### Relative activity and kinetic parameters

The relative activity of *B. licheniformis *mannan endo-1,4-β-mannosidase against konjac glucomannan, 1,4-β-D-mannan, locust bean gum, guar gum and copra meal was determined by pre-incubating 5 mg/ml of each substrate in 0.1 M phosphate buffer pH 7.0 at 50°C for 30 min with constant agitation using a Thermomixer comfort (Eppendorf AG, Hamburg, Germany). After adding the purified enzyme (276 ng), the reaction was incubated at 50°C with shaking for 5 min, and then terminated by boiling for 10 min. The release of reducing sugars was detected by the DNS method as described above. Relative mannan endo-1,4-β-mannosidase activities against various substrates were calculated by converting A_540 _to μmoles of mannose released.

For determination of the kinetic parameters, various concentrations of different substrates in 0.1 M phosphate buffer pH 7.0 [konjac glucomannan, low viscosity (3-19.5 mg/ml); 1,4-β-D-mannan (3-30 mg/ml); locust bean gum, low viscosity (3-39 mg/ml)] were incubated with the purified mannan endo-1,4-β-mannosidase (138 ng) at 50°C for 5 min. The Vmax and Km values were calculated by non-linear regression analysis, using the GraphPad Prism software (GraphPad Software Inc., San Diego, CA).

Mannose, konjac glucomannan (low viscosity) and 1,4-β-D-mannan (prepared by controlled hydrolysis of carob galactomannan, DP < 15) were purchased from Megazyme International (Bray, Ireland). Locust bean gum (LBG) was isolated from Ceratonia siliqua seeds (Sigma-Aldrich). Low-viscosity locust bean gum was prepared according to a previously published protocol [18]. Guar gum was purchased from Sigma-Aldrich, while copra meal was bought from a local market in Nakhon Ratchasima province, Thailand.

### Thin-layer chromatography

Hydrolysis of 15 mM substrates (manno-oligosaccharides M2-M6), 0.1 mg LBG (high viscosity) and 0.1 mg of 1,4-β-D-mannan by mannan endo-1,4-β-mannosidase was carried out in a 30-μl reaction mixture, containing 0.1 M phosphate buffer, pH 7.0, and 13.8 ng (for M2 - M6), 276 ng (for LBG) or 2.76 μg (for 1,4-β-D-mannan) of purified enzyme. The reaction mixture was incubated at 50 °C with shaking for 2, 5, 10, 15, 30, 60 min, and 12 h prior to termination of the hydrolysis reaction by boiling for 5 min. A sample of each reaction mixture was applied five times (one μl each) to a silica TLC plate (6.0 × 10.0 cm), and then chromatographed twice (2 h each) using a mobile phase containing n-propanol: ethanol: water (7:1:2) (v/v), followed by spraying with 5% sulphuric acid and heating at 180°C for 3 min. A mixture of M1-M6 (5 nmol each) was used as standard. Manno-oligosaccharides (M2-M6), galacto-manno-oligosaccharides (OGM2 and OGM3), mannose and galactose were from Megazyme, and locust bean gum was from Sigma-Aldrich. Silicagel 60 F254 aluminum sheet, n-propanol and ethanol were purchased from Merck (Damstadt, Germany).

## Competing interests

The authors declare that they have no competing interests.

## Authors' contributions

CS performed amino acid sequence analysis, purified and analyzed the enzyme. BB expressed and characterized the enzyme properties. DH supervised enzyme characterization, co-designed experiments, evaluated the data, and edited the manuscript. MY conceived of the study, participated in cloning and expression of the enzymes, and wrote the manuscript. All authors read and approved the final manuscript.

## Supplementary Material

Additional file 1 **Properties of various mannan endo-1,4-β-mannosidases; pdf format; A summary of properties of different mannan endo-1,4-β-mannosidases, i.e. Source, GH family, pH and temperature optima, yield, stability, specific activity, kinetic parameter using LBG as substrate, and reference **[38-50]. Click here for file
