# Supplementary material for: Efficient recombinant expression and secretion of a thermostable GH26 mannan endo-1,4-β-mannosidase from Bacillus licheniformis in Escherichia coli
Source: Microb Cell Fact. 2010 Apr 11;9:20. doi: 10.1186/1475-2859-9-20 (PMC2868798; doi:10.1186/1475-2859-9-20)
Supplement: Additional file 1 — Properties of various mannan endo-1,4-β-mannosidases; pdf format; A summary of properties of different mannan endo-1,4-β-mannosidases, i.e. Source, GH family, pH and temperature optima, yield, stability, specific activity, kinetic parameter using LBG as substrate, and reference [38-50]. [file 1475-2859-9-20-S1.PDF]

Additional File 1 - Properties of various mannan endo-1,4-β-mannosidases

Organism	Family GH [a]	native/recombinant [b]	Activity (Yield) [c]	K_m (mg/ml) / V_{max} (U/mg)	Optimal pH/temperature	Stability	Ref
Aspergillus aculeatus MRC11624	5	Recombinant (*Saccharomyces cerevisiae*)	64.6 U/mg (31.3 U/ml)	K_m 0.3/ V_{max} 82	pH 3/ Temp 60°C	90% activity at 50°C, pH 4 - 6 after 24 h	[38]
Aspergillus fumigatus IMI 385708	-	Native	~ 600 U/mg	K_m ~ 3/ V_{max} ~2,000 (low viscosity LBG)	pH 4.5/ Temp 65°C	Stable at pH 4.5 - 8.5 for 5 h up to 55 °C	[39]
Aspergillus niger ATCC-46890	-	Native	231.6 U/mg	ND	pH 3.5	Stable at pH 3.5 - 7.5 for 24 h at 50°C	[40]
Aspergillus niger BK01	5	Recombinant (*Pichia pastoris*)	2570 U/mg (243 µg/l)	K_m 2.0/ V_{max} 373	pH 4.5/ Temp 80°C	Stable at pH 4.0 for 80 h up to 50 °C	[28]
Aspergillus sulphureus	5	Recombinant (*Pichia pastoris*)	366 U/mg	K_m 0.93/ V_{max} 3.8	pH 2.4/ Temp 50°C	Stable at pH 2.2 - 8.0 at 40°C for 1 h	[41]

Organism	Family GH [a]	native/recombinant [b]	Activity (Yield) [c]	K_m (mg/ml) /V_{max} (U/mg)	Optimal pH/temperature	Stability	Ref
Aspergillus sulphureus	5	Recombinant (*E. coli*)	208 U/mg (found in inclusion body)	K_m 1.38/ V_{max} 73	pH 2.4/ Temp 50°C	80% activity at 40 °C , pH 2.4 after1 h<br>No activity at 60 °C after 30 min.	[42]
Alkaliphilic *Bacilllus* sp. N16-5	5	Recombinant (*Pichia pastoris*)	32.2 U/ml	ND	pH 10/ Temp 70°C	90% of activity after incubation at 70°C for 1h<br>73% of activity after incubation at<br>pH 12 at 50°C for 1 h	[19]
Bacillus circulans CGMCC 1416	5	Recombinant (*E. coli*)	481.55 U/mg	ND	pH 7.6/ Temp 58°C	ND	[25]
Bacillus licheniformis	-	Native	4,341 U/mg	ND	pH 7.0/ Temp 60°C	Stable at 50°C and pH 6.0 for 6 h	[12]
Bacillus licheniformis DSM13 and DSM8785	26	Recombinant (*E. coli*)	1,560 U/mg<br>~ 60 U/ml	ND	pH 6 - 7/ Temp 50˚C	stable within pH 5 - 12 at 50°C for 30 min, and pH 6 - 9 at 50°C for 24 h	This study

Organism	Family GH [a]	native/recombinant [b]	Activity (Yield) [c]	K_m (mg/ml) /V_{max} (U/mg)	Optimal pH/temperature	Stability	Ref
Bacillus subtilis B36		Recombinant (*E. coli*)	927.84 U/mg		pH 6.4/ Temp 50˚C	Stable at 60 °C	[20]
Bacillus subtilis NM-39	26	Recombinant (*E. coli*)	4.9 U/ml	ND	ND	ND	[21]
Bacillus subtilis NRRL 356	-	Native	106.2 U/ml	ND	pH 6.5 - 6.8/	100% of activity after incubation at 65°C for 3 h	[11]
Bacillus subtilis WL-3	26	Recombinant (*E. coli*)	5,900 U/mg		pH 6.0/ Temp 60°C	No activity after incubation at 4°C in the presence of 3mMFe^{2+} for 24 h	[22]
Bacillus subtilis WY34		Native	8,302.4 U/mg	K_m 7.6±0.2/ V_{max} 970±10.3	pH 6.0/ Temp 65˚C	pH 5.5-10.1 for 30 min at 50°C/ 60˚C, for 30 min pH 6.0	[29]
Bacillus subtilis Z-2	26	Recombinant (*E. coli*)	~ 40 U/ml	K_m 10.2/ k_{cat} 3672	pH 4.5/ Temp 60°C	>80% of activity after incubation between 50 - 80°C for 15 min >80% of activity after incubation between pH 2.5 - 8.5 for 15 min	[19, 23]

Organism	Family GH [a]	native/recombinant [b]	Activity (Yield) [c]	K_m (mg/ml) /V_{max} (U/mg)	Optimal pH/temperature	Stability	Ref
Bacillus. stearothermophilus	5	Both (the activity is comparable)	100 U/mg	K_m ~2/ V_{max}~400	pH 5.5 - 7.5	>90% of activity after incubation at 70°C for 24 h	[43]
Caldocellum saccharolyticum	5	Recombinant (*E. coli*)	ND	ND	pH 6/ Temp 80°C	Stable at 80°C for 1 h	[44]
Mytilus edulis (blue mussel)	5	Recombinant (*Pichia pastoris*)	45.6 U/mg	ND	pH 5.2/ Temp 50°C	Stable between pH 4.0 - 9.5 at room temp for 24 h	[45, 46]
Rhodothermus marinus ATCC 43812	26	Recombinant (*E. coli*)	ND	ND	pH 5.4/ Temp 85°C	>70% after 1 h at 70°C	[31]
Thermotoga neapolitana 5068	-	Native	3.8 U/mg	K_m 0.55/ V_{max} 3.8	pH 6.9/ Temp 90-92°C	Half life of 34 h at 85°C, 13 h at 90°C, and 35 min at 100 °C	[30]
Trichoderma reesei	5	Native	85.8-111.6- U/mg	ND	pH 3.5 - 4.0/ Temp 70°C	Stable for 24 h at 30°C, pH 5.3	[47, 48]
Trichoderma reesei	5	Recombinant (*Pichia pastoris*)	12.5 U/ml		pH 5.0/ Temp 80°C	>50% at 70°C after 30 min Stable at pH 5.0-6.0	[49]
Vibrio sp. MA-138	5	Native	ND	ND	pH 7.5/ Temp 45°C	-	[50]

*Locust bean gum was used for measuring β-mannanase activities and determination of kinetic parameters

[a] glycosyl hydrolase (GH) family, the family is only assigned to enzymes with known amino acid sequence

[b] expression system of the recombinant enzyme is indicated in parenthesis

[c] specific activity is indicated as U of mannan endo-1,4-β-mannosidase activity per mg protein. In some case, the yield is indicated as U per ml of culture media in parenthesis.

ND = not determine
